# Pan-cancer analysis of the intervertebral-disc-degeneration-related innate immunity gene NAIP

**DOI:** 10.1371/journal.pone.0286647

**Published:** 2023-06-02

**Authors:** Wen-Bin Xu, Vit Kotheeranurak, Ding-Qiang Chen, Nai-Kun Sun, Di-Xin Cai, Chien-Min Chen, Guang-Xun Lin, Gang Rui

**Affiliations:** 1 Department of Orthopedics, The First Affiliated Hospital of Xiamen University, School of Medicine, Xiamen University, Xiamen, China; 2 Department of Orthopedics, Faculty of Medicine, Chulalongkorn University and King Chulalongkorn Memorial Hospital, Bangkok, Thailand; 3 Center of Excellence in Biomechanics and Innovative Spine Surgery, Chulalongkorn University, Bangkok, Thailand; 4 The Third Clinical Medical College, Fujian Medical University, Fuzhou, Fujian, China; 5 Division of Neurosurgery, Department of Surgery, Changhua Christian Hospital, Changhua, Taiwan; 6 Department of Leisure Industry Management, National Chin-Yi University of Technology, Taichung, Taiwan; 7 School of Medicine, Kaohsiung Medical University, Kaohsiung, Taiwan; Southern Medical University, CHINA

## Abstract

**Background:**

Intervertebral disc degeneration (IDD) is a progressive chronic condition that commonly causes low back pain. Cancer is among the primary reasons for deaths worldwide. Our purpose was to identify the characteristic genes of IDD and explore the potential association between IDD and cancer.

**Methods:**

Immune cell infiltration and differentially expressed analysis were conducted utilizing data from the GSE124272 database. Enrichment analysis of differentially expressed genes (DEGs) was performed to explore the possible mechanisms underlying IDD development. Moreover, weighted gene correlation network analysis (WGCNA) was applied to select IDD-related hub genes. The immune-related key genes were determined by intersecting DEGs, IDD-related hub genes, and immune genes. Subsequently, machine learning models based on these genes were built to identify and verify the characteristic genes. RNA sequencing and clinical data of 33 carcinoma categories were obtained from the Cancer Genome Atlas (TCGA). The association between NAIP expression and prognosis was calculated using the Kaplan-Meier analysis. To gain a deeper understanding of the impact of NAIP in tumor immunotherapy, the association between NAIP and immune infiltration and two immunotherapeutic biomarkers were explored. Ultimately, the association between NAIP and immunotherapeutic response was investigated utilizing two independent cohorts.

**Results:**

NAIP was identified as an immune-related characteristic gene between IDD and normal intervertebral disc tissue. In certain carcinoma categories, NAIP expression levels were elevated (4/33) and significantly correlated to the respective tumor stage (4/21). Survival analysis revealed that the expression levels of NAIP have prognostic significance in different cancer types. Generally, NAIP presented a strong association with immune cell infiltration and modulators. NAIP may influence immunotherapy effects through tumor mutational burden and microsatellite instability. No remarkable association between NAIP and immunotherapy response was found in either cohort.

**Conclusion:**

Our study is the first to identify NAIP as an immune-related characteristic gene. Pan-cancer analysis revealed that NAIP could serve as a novel clinical prognostic marker and therapeutic target for a variety of carcinoma categories, reducing the risk of IDD in tumor patients.

## Introduction

Low back pain is a broad and complicated clinical condition that affects 80% of the population worldwide [1]. Numerous factors can cause low back pain; however, proof of nerve compression cannot be found in over 40% of patients with chronic low back pain [2]. Hence, intervertebral disc degeneration (IDD) is regarded as one of the major factors that cause back pain and stiffness [3]. IDD is recognized as a global health issue due to the enormous pressure on the healthcare system and the subsequent economic burden [4]. Although several familiar elements, such as mechanical stimulation, degeneration, and inflammation [5–7], promote the progression of IDD, the detailed process of IDD occurrence remains unclear. The current treatment of IDD mainly includes conservative and surgical treatment, which provide symptomatic and supportive measures, but cannot reverse IDD and reconstruct the mechanical function of the spine [8]. Cancer occurrence is a complicated process consisting of multiple risk factors, rendering this disease as the leading cause of death worldwide [9].

There is growing evidence that immunity affects IDD and cancer significantly [10, 11]. A previous study suggested that disruption of the blood nucleus pulposus barrier triggers immune reactions via the NP cells, which have been found to be important factors in the development of IDD [12]. In addition, proinflammatory cytokines generated by immune cells, such as interleukin-1β and tumor necrosis factor-alpha, have been found to induce degeneration and apoptosis of NP cells by activating the β-catenin [5, 13]. However, the immune landscape and regulatory mechanism of immune cells in IDD remain unknown. Immune infiltrates in the tumor microenvironment (TME) play an essential role in tumor progression and influence the prognosis of patients with cancer [14]. Therefore, a comprehensive analysis of tumor-infiltrating immune cells will help elucidate the mechanisms of tumor immune evasion, laying the foundation for the exploration of novel treatment approaches [14].

**Fig 1:**
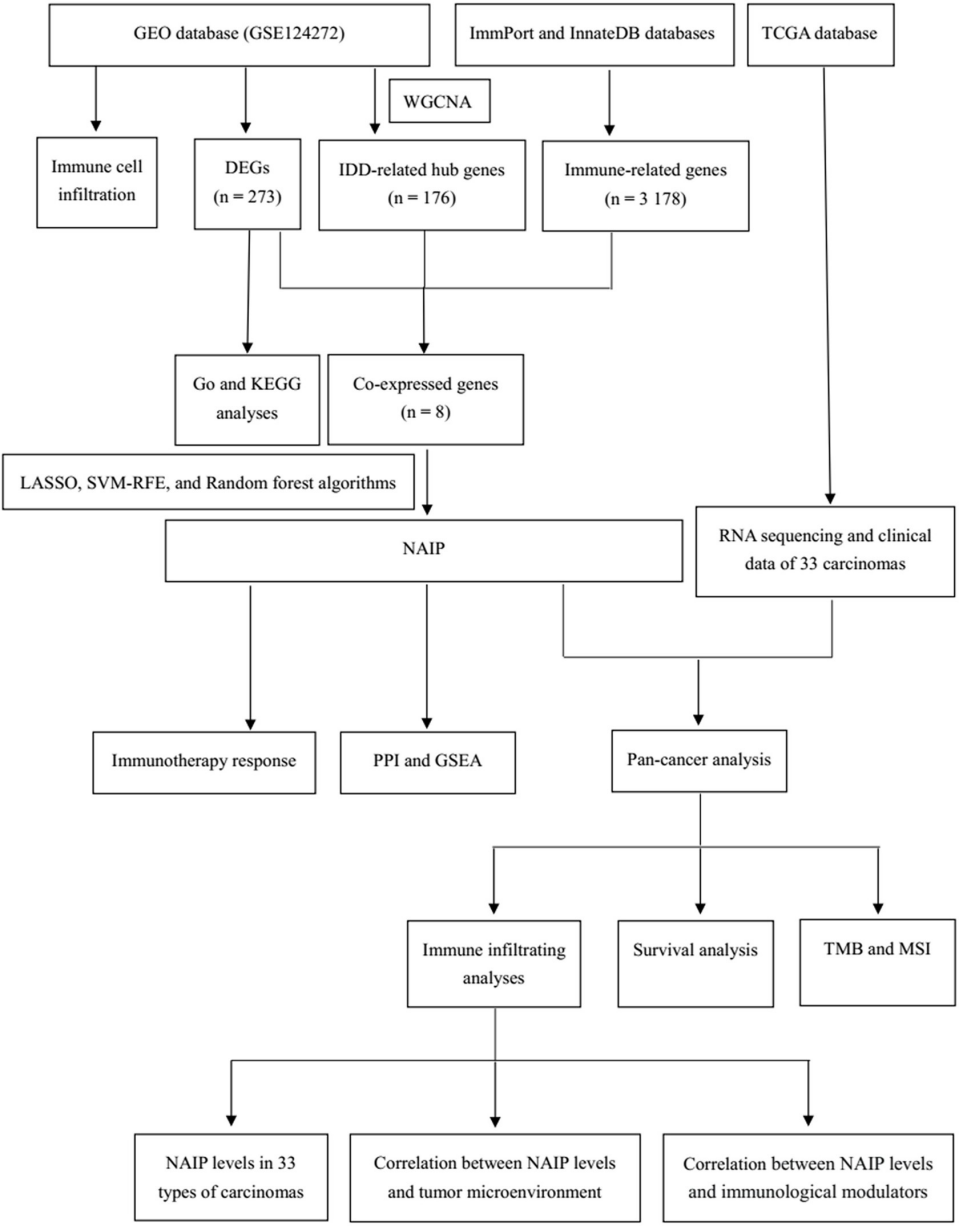
Flowchart of this study.

Our purpose was to identify IDD-related innate immunity genes utilizing bioinformatics analysis and explore their roles in diverse cancer types with the hope to provide new perspectives on the link between IDD and cancer and reveal possible therapeutic targets for IDD patients with carcinoma. Fig 1 shows the flowchart of the entire procedure.

## Methods

### Microarray data

Microarray data involving eight healthy volunteers and eight IDD patients were extracted from the Gene Expression Omnibus (GEO) database (GSE124272) [15]. The RNA sequencing and clinical data for 33 diverse carcinoma categories were obtained from the TCGA database [16]. In addition, 3178 unique genes related to the immune were acquired from the Immunology Database and Analysis Portal (ImmPort) and InnateDB databases [17, 18]. Finally, two tumor immunotherapeutic cohorts (GSE78220 and GSE67501) were acquired from the GEO database [15].

### Estimation of immune cell infiltration

The CIBERSORT deconvolution algorithm was applied to estimate the abundance of 22 kinds of immune cells and evaluate the percentage of different immune cells in 16 samples among GSE124272 through expression values. Furthermore, we estimated discrepancies between IDD and control samples. Finally, the stacked bar plot, correlation heatmap, and violin diagram were visualized by “corrplot” and “vioplot” packages. The selection criteria were adjusted *P* value < 0.05.

### Differential expression analysis

Differentially expressed genes (DEGs) of GSE124272 were selected by using the Bioconductor Limma package, with *P* < 0.05 and |log2(fold-change)| > 1.00 [19]. Furthermore, the volcano and heatmap of DEGs were visualized by the pheatmap package.

### Functional and pathway correlation analysis

To explore the role of DEGs, the Gene Ontology (GO) and Kyoto Encyclopedia of Genes and Genomes (KEGG) analyses were implemented utilizing the clusterProfiler package [20]. Both the *p* and *q* values of the GO analysis were < 0.05. The q-value < 1.00 of KEGG analysis was considered significant enrichment.

### Weighted Gene Correlation Network Analysis (WGCNA)

WGCNA R package is a biological analysis approach for clustering genes into modules according to interconnectivity, allowing the detection of hub genes through the most significant module [21]. Therefore, we established a co-expression network based on all GSE124272 genes utilizing the WGCNA. First, the sample cluster analysis was conducted using the *hclust* function to eliminate outliers. Then, the soft thresholding power value was identified using the *pickSoftThreshold* function to obtain a nearly scale-free network topology [21, 22]. Furthermore, the whole genes were sorted into different modules according to a dynamic tree cutting arithmetic with at least 100 genes in the module, and modules with significantly related eigengenes (correlation > 0.25) were combined [23]. Finally, the module with p < 0.05 and the highest correlation coefficient was chosen for further investigation.

### Identification of immune-related key genes

Following identification of the key module through WGCNA, the association between innate immune genes and IDD progression was explored. The immune-related key genes were determined by intersecting three parts (DEGs, Hub genes from WGCNA, and Immune genes downloaded from databases) using the Venn diagram.

### Establishment of machine learning to verify the reliability of key genes

The least absolute shrinkage and selection operator (LASSO) logistic regression [24], support vector machine-recursive features elimination (SVM-RFE) [25], and random forest algorithms were performed to validate the robustness of the selected key genes. The LASSO algorithm was implemented with the “glmnet” package [26]. SVM-RFE analysis was performed by the e1071 package [27]. Furthermore, the random forest algorithm was conducted by the R package. Ultimately, three-fold cross-verification was conducted to obtain the best predicting results using the Venn diagram.

### Assessment of diagnostic value of key genes

To validate whether key genes could discriminate between IDD and control samples in GSE124272, the receiver operating characteristic curve assessed the diagnostic value of key genes using the pROC R package [28].

### Survival analysis

The Kaplan-Meier survival analysis was utilized to forecast the correlation between the overall survival (OS) of patients and NAIP expression levels in 33 diverse cancer types.

### Immune infiltrating analysis associated with NAIP

The CIBERSORT algorithm was utilized to quantify the abundance of 22 kinds of immune cells in 33 diverse cancer types, and the correlation between NAIP levels and these cells was evaluated [29]. A correlation coefficient > 0.4 was considered significant. Subsequently, the tumor immune microenvironment, including stromal content (StromalScore), immune infiltration (ImmuneScore), and combined (ESTIMATEScore) score of each tumor sample, were estimated via the ESTIMATE R package [30], and the association between NAIP and tumor immune microenvironment was assessed by R. A correlation coefficient > 0.6 was considered significant. Moreover, the potential association between NAIP levels and immunological modulators (immune inhibitors, immune stimulators, and MHC molecules) was investigated using the TISIDB database (http://cis.hku.hk/TISIDB/).

### Mutation and microsatellite instability

Currently, tumor mutational burden (TMB) and Microsatellite instability (MSI) are novel genetic biomarkers forecasting the efficacy of immunotherapy. The relationship between NAIP and novel dynamic biomarkers of the immune checkpoint blockade (TMB/MSI) was predicted by R (Spearman’s rank correlation test).

### PPI network and enrichment analysis

Protein-protein interaction (PPI) network with NAIP as the core was constructed using the GeneMANIA online tool (http://genemania.org/) [31]. Furthermore, the Metascape (http://metascape.org/) was applied to perform enrichment analysis of genes from the PPI network [32]. Additionally, the biological signaling pathways between high and low NAIP expression groups were performed using gene set enrichment analysis (GSEA) [33].

### Immunotherapy response

Two immunotherapy cohorts were used for immunotherapy response analysis, and discrepancies between the response (responding to immunotherapy) and non-response (non-responding to immunotherapy) groups were determined by R (Wilcoxon test).

## Results

### Immune cell infiltration

**Fig 2:**
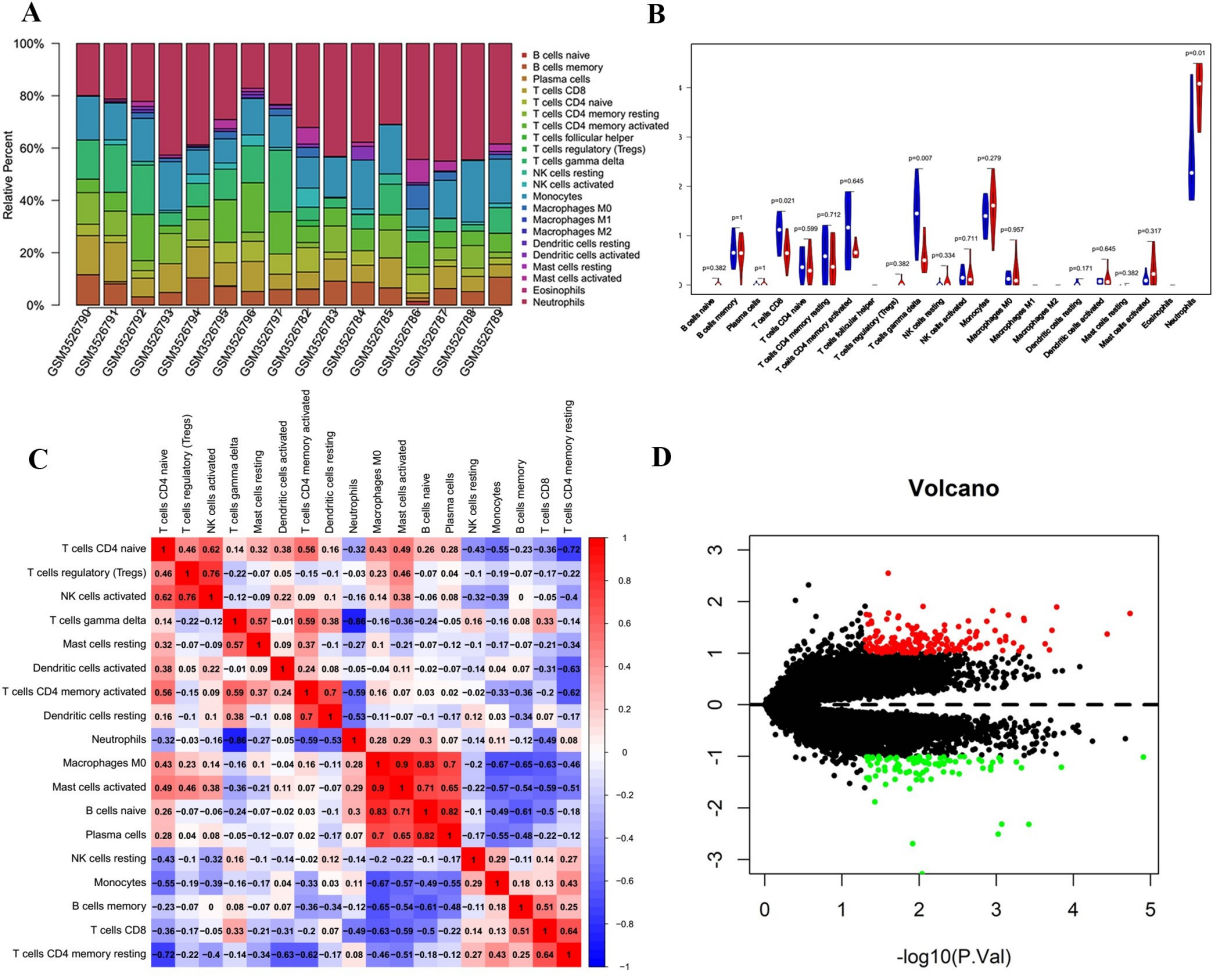
Visualization of immune cell infiltration analysis. (A) Stacked bar plot of the percentage of infiltrating immune cells in each individual. (B) Violin diagram of the abundance of infiltration by 22 immune cell subsets between IDD and control groups. Red and blue colors represent IDD and control groups, respectively. (C) Correlation matrix of 22 immune cell type proportions. Red represents positive correlation, whereas blue represents negative correlation. (D) Volcano map of DEGs, where red represents upregulated genes and green represents downregulated genes.

The specific immune cell types infiltrating into the IDD tissues were analyzed utilizing the CIBERSORT algorithm. The stacked bar plot showed the percentage of infiltrating immune cells (Fig 2A). The violin diagram demonstrated that CD8 and γ/δ T cell levels were remarkably lower in IDD, whereas neutrophil expression was remarkably higher in IDD (Fig 2B). Fig 2C demonstrates a significant positive correlation between activated Mast cells and Macrophages M0, and a significant negative correlation between γ/δ T cells and neutrophils.

### Identification of DEGs

The microarray dataset (GSE124272) was applied to select DEGs in IDD samples. Under the criteria set in advance, 273 genes (182 upregulated and 91 downregulated) were identified as DEGs (Fig 2D).

### Functional and pathway enrichment analyses

**Fig 3:**
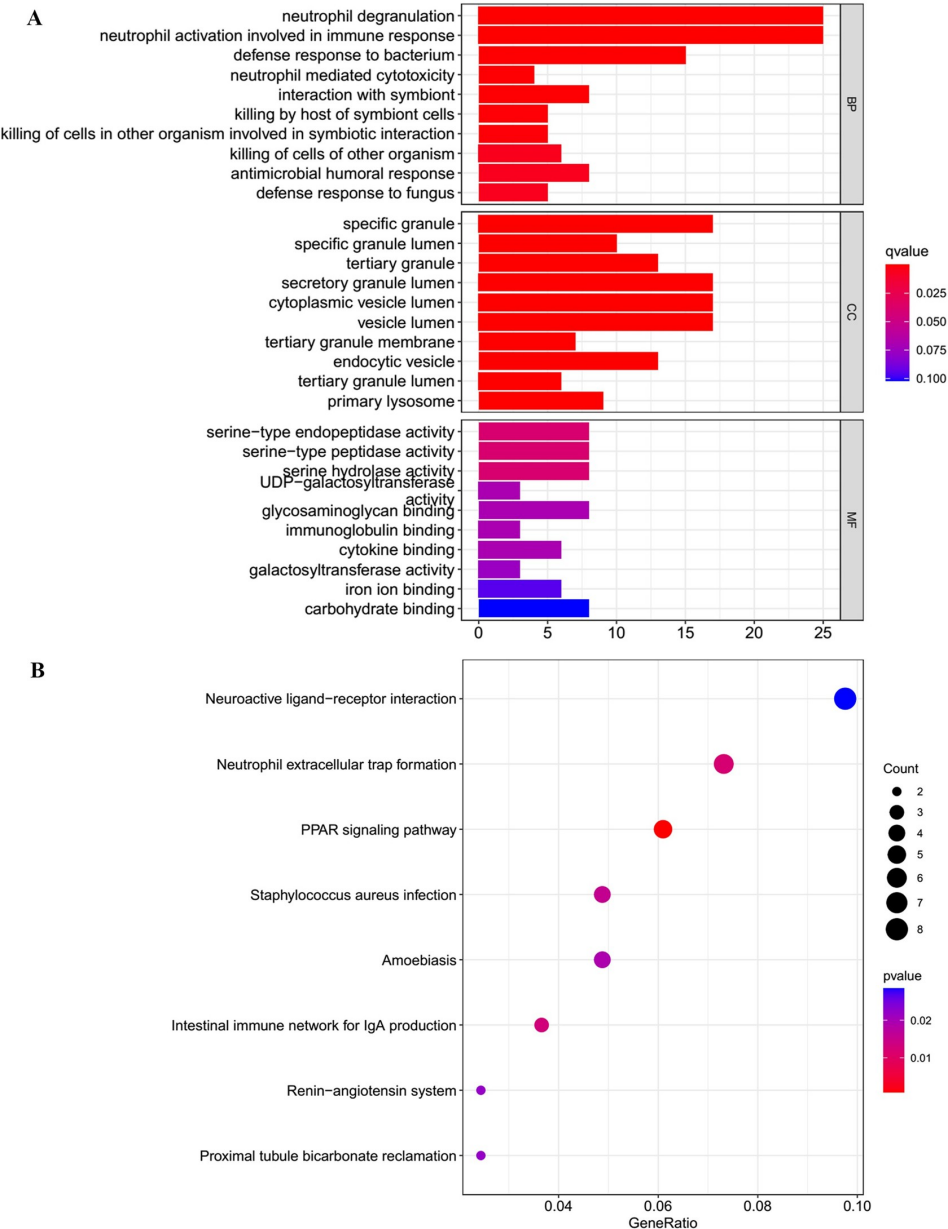
GO and KEGG enrichment analysis. (A) Bar plot of BPs, CCs, and MFs from GO analysis. (B) Dot plot of KEGG pathways analysis.

GO analysis demonstrated that DEGs were clustered in 17 biological process (BP) terms (mainly including neutrophil degranulation and neutrophil activation involved in immune response), 29 cellular component (CC) terms (primarily including specific granule, specific granule lumen, tertiary granule, secretory granule lumen, cytoplasmic vesicle lumen, and vesicle lumen), and three molecular function (MF) terms (including serine-type endopeptidase activity, serine-type peptidase activity, and serine hydrolase activity) (Fig 3A). Moreover, KEGG demonstrated that such genes were centred in eight relevant pathways (primarily including the PPAR signaling pathway and neutrophil extracellular trap formation) (Fig 3B).

### Establishing weighted gene correlation network

**Fig 4:**
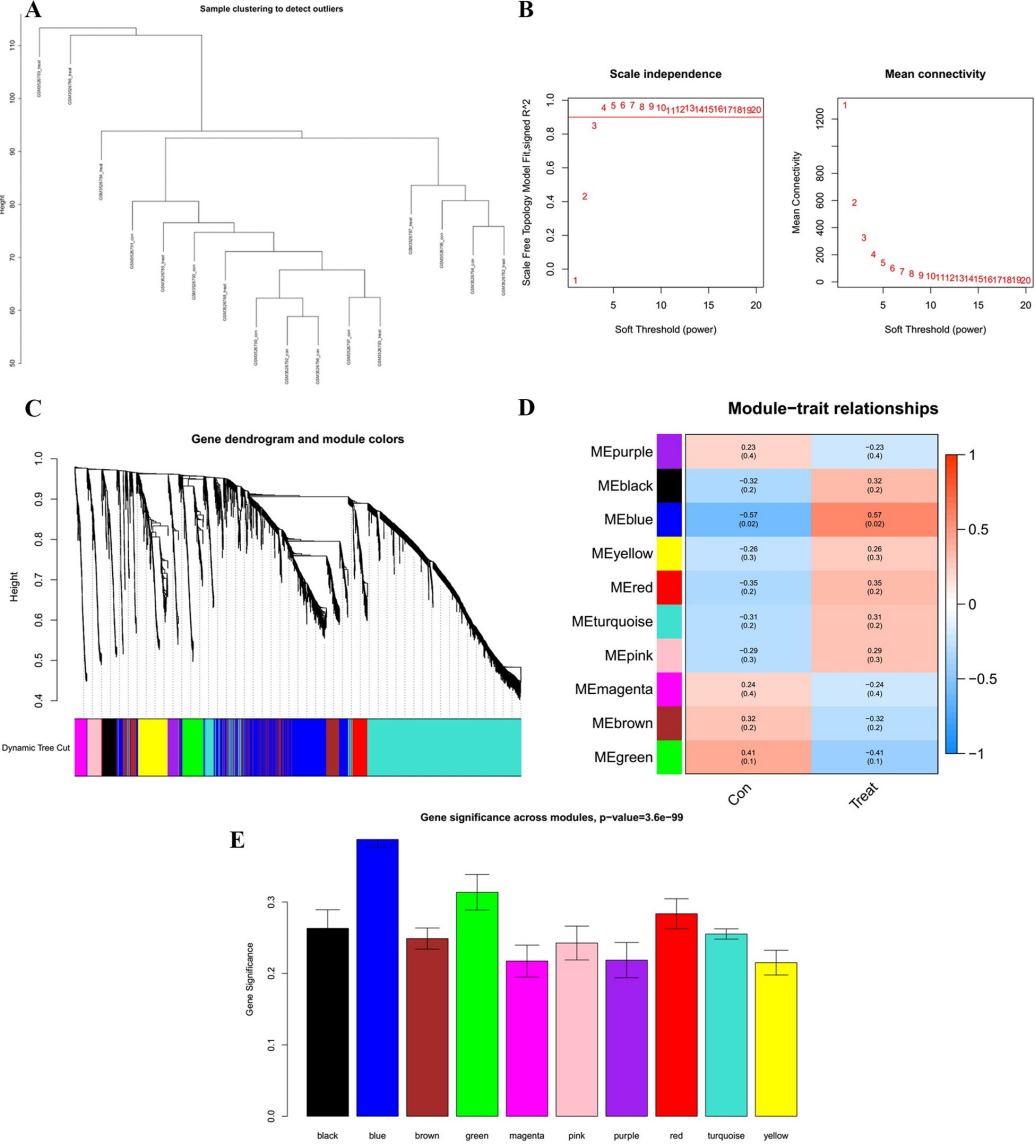
Identification of the key module related to IDD through WGCNA. (A) Sample cluster analysis identified the outlier samples in the GSE124272 dataset. (B) Determination of the optimal soft threshold to conform to the scale-free distribution. (C) Dendrogram of genes clustered based on the highly correlated eigengenes (correlation above 0.3). (D) Heatmap of the correlation between module eigengenes and clinical traits. (E) Histogram of gene significance across modules.

First, whole genes from GSE124272 were selected for WGCNA analysis. The heterogeneity of 16 samples was detected, and outliers were checked and removed. As shown in Fig 4A, all samples were included. Then, the soft threshold power of β (scale-free R^2^ = 0.90) was set to 4, satisfying the distribution of a scale-free network (Fig 4B), and the modules with eigengenes correlation above 0.3 were merged (Fig 4C). Consequently, 10 modules were identified for further exploration (Fig 4D). After analyzing the correlation between modules and traits, we found that the blue module was remarkably associated with IDD (Fig 4D). Fig 4E also confirmed the reliability of our findings. Hence, 1035 genes (176 hub genes) from the blue module were identified as IDD-associated genes.

### Identification and validation of hub immune-related genes

**Fig 5:**
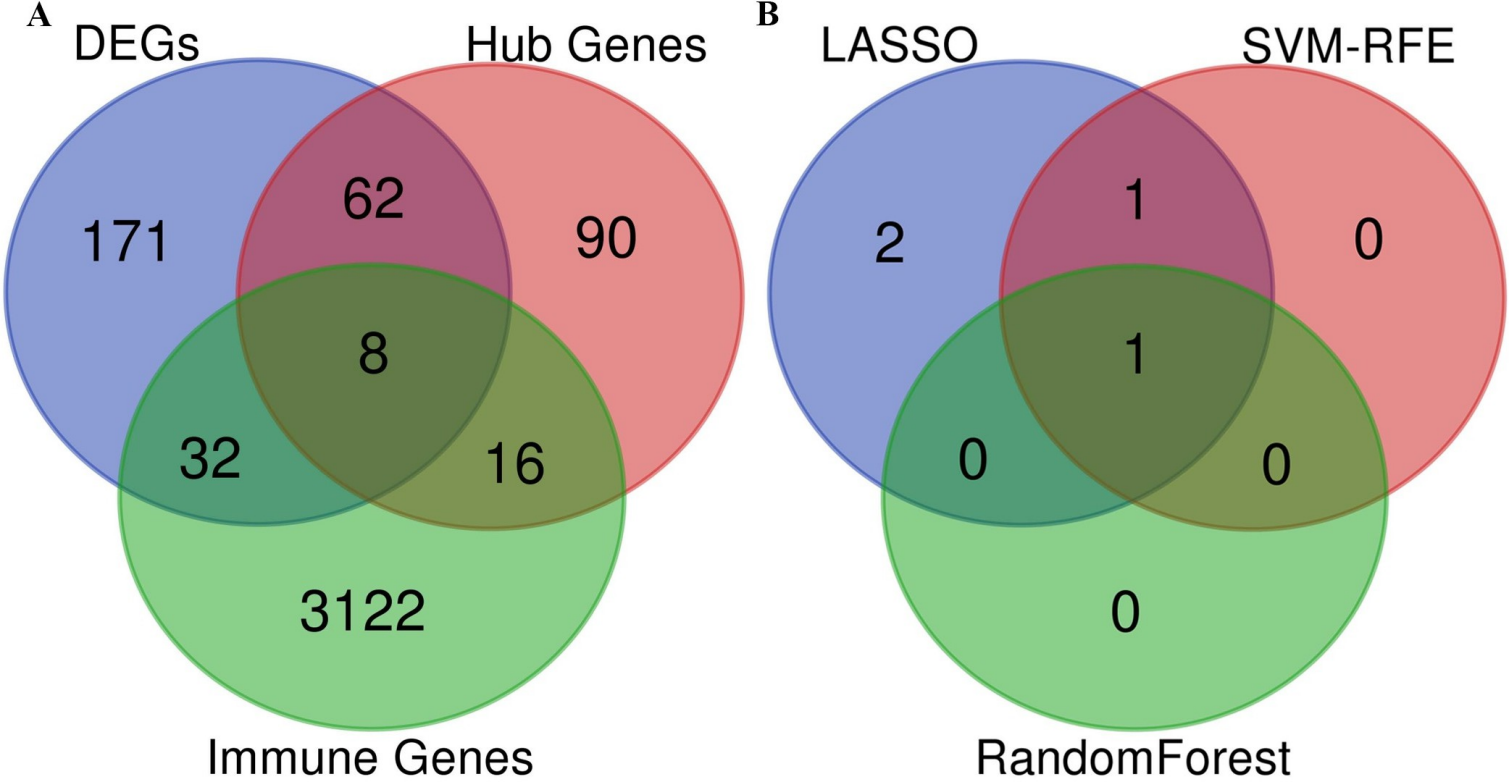
(A) Venn diagram revealed that eight genes were commonly expressed in three parts. (B) Venn diagram showed the overlapped gene obtained by the LASSO algorithm, VM-RFE algorithm and random forest algorithm.

**Fig 6:**
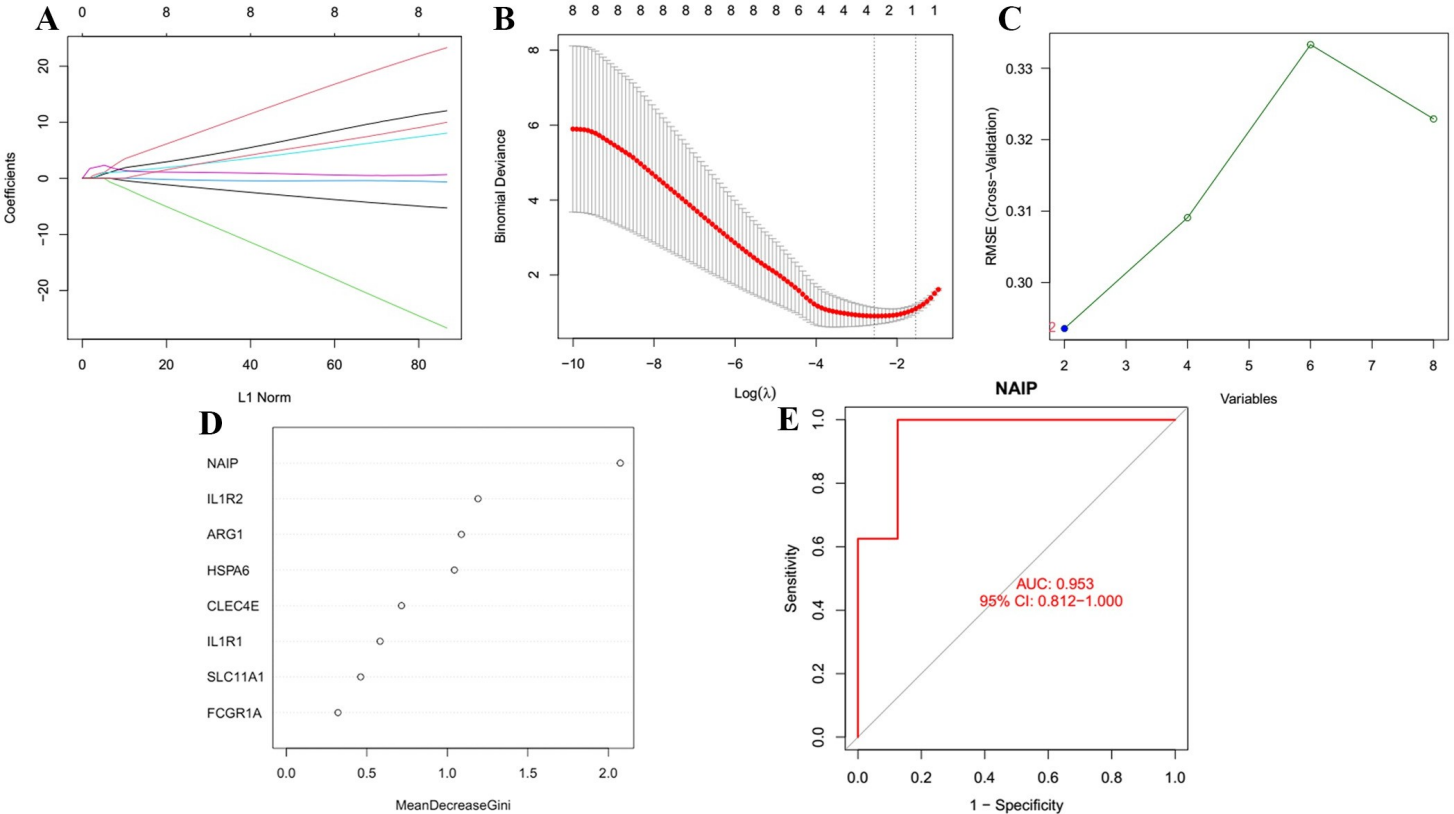
(A, B) LASSO regression to screen for characteristic genes. (C) SVM to screen for characteristic genes. (D) Random forest algorithm to screen for characteristic genes. (E) ROC curve analysis of the NAIP for predicting IDD.

Considering the significant role of immune response in IDD, 3178 immune-related genes were acquired from the ImmPort and InnateDB databases. Eight co-expressed genes (CLEC4E, NAIP, SLC11A1, FCGR1A, IL1R1, HSPA6, ARG1, and IL1R2) were identified by intersecting 273 DEGs, 176 hub genes from the blue module, and 3178 immune-related genes (Fig 5A). Subsequently, machine learning models (LASSO, SVM-RFE, and random forest algorithms) based on the eight co-expressed genes were built to validate their prediction power. The LASSO algorithm determined four characteristic genes (CLEC4E, NAIP, IL1R1, and HSPA6) (Fig 6A and 6B), the SVM-RFE algorithm identified two characteristic genes (NAIP and HSPA6) (Fig 6C), and the random forest algorithm identified only one characteristic gene (NAIP) (Fig 6D). After validation, one diagnostic gene (NAIP) was identified (Fig 5B). Furthermore, the AUC value of NAIP was > 0.7 in the GSE124272, indicating that NAIP could be used as a diagnostic biomarker (Fig 6E).

### Clinical landscape of NAIP expression in carcinomas

**Fig 7:**
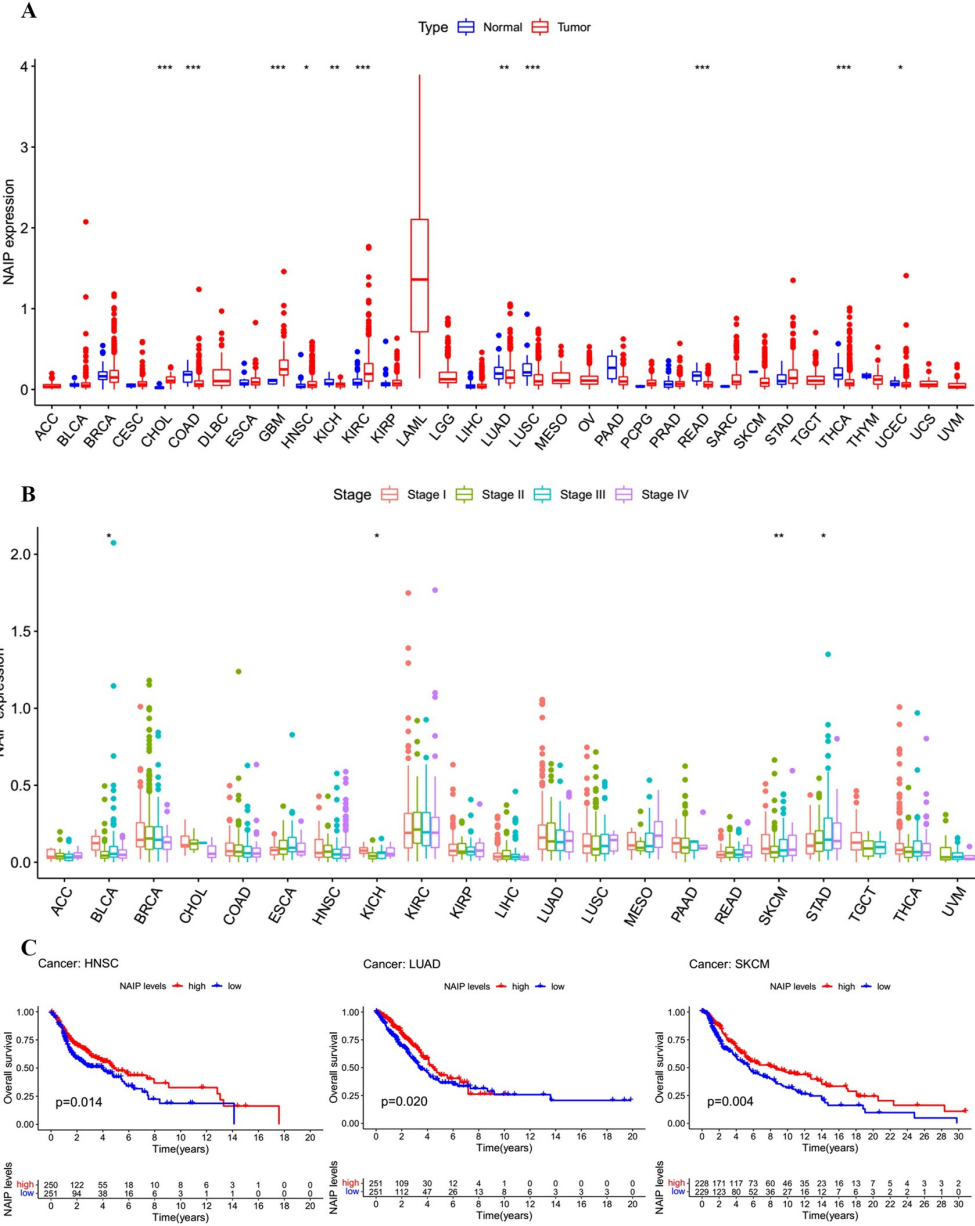
(A) Expression levels of NAIP in 33 cancers. (B) The correlation between tumor stage and NAIP expression levels. (C) Relationship between NAIP expression levels and prognosis (Kaplan-Meier curves for OS). “*” indicates *p* < 0.05, “**” indicates *p* < 0.01 and “***” indicates *p* < 0.001.

S1 Table demonstrates 33 types of carcinomas included in this study. We utilized the TCGA database to calculate the NAIP levels in tumor and normal tissues, revealing that NAIP was differentially expressed in 11 of the 33 carcinomas (CHOL, COAD, GBM, HNSC, KICH, KIRC, LUAD, LUSC, READ, THCA, and UCEC) (Fig 7A). Also, NAIP expression levels were remarkably related to the tumor stage of certain carcinomas (BLCA, KICH, SKCM, and STAD) (Fig 7B).

Furthermore, the relationship between the survival time and NAIP expression levels was investigated. The outcomes demonstrated that the NAIP expression levels was positively associated with OS in SKCM (*p* = 0.004), LUAD (*p* = 0.020), and HNSC (*p* = 0.014) (Fig 7C). Overall, the survival analysis revealed that levels of NAIP in different cancer types had significant prognostic implications.

### Association between NAIP expression and immune-related factors

**Fig 8:**
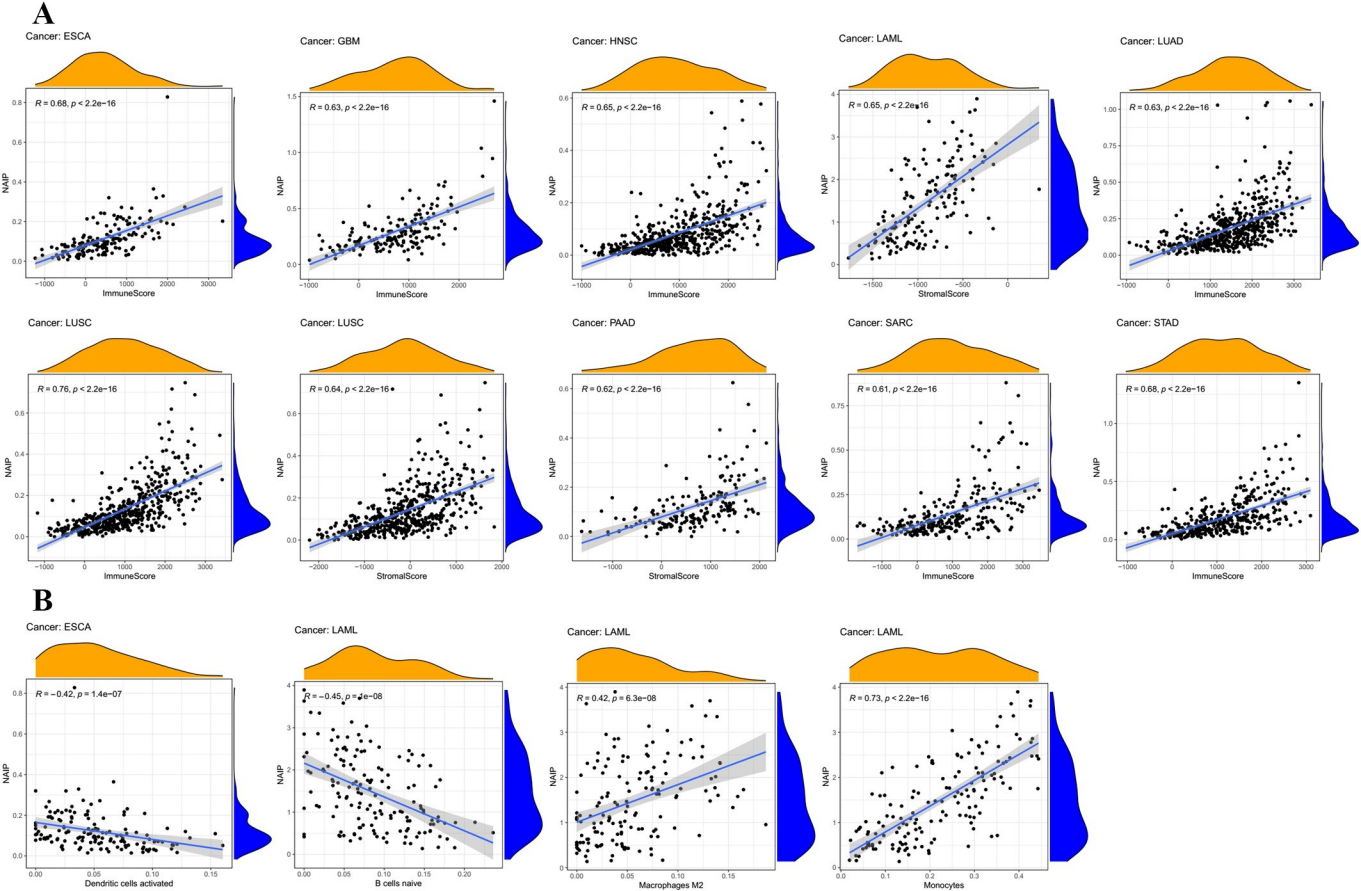
(A) Correlation between NAIP expression and ESTIMATE score (including stromal score and immune score). (B) Correlation between NAIP expression and immune infiltration.

**Fig 9:**
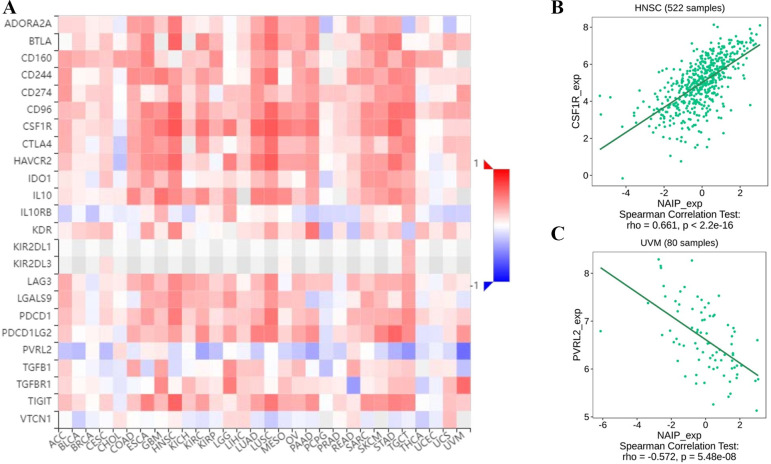
The expression correlation between NAIP and immune inhibitors. Red indicates positive correlation whereas blue indicates negative correlation. (A) Dotplot of the strongest positive association. (B) Dotplot of the strongest negative association.

**Fig 10:**
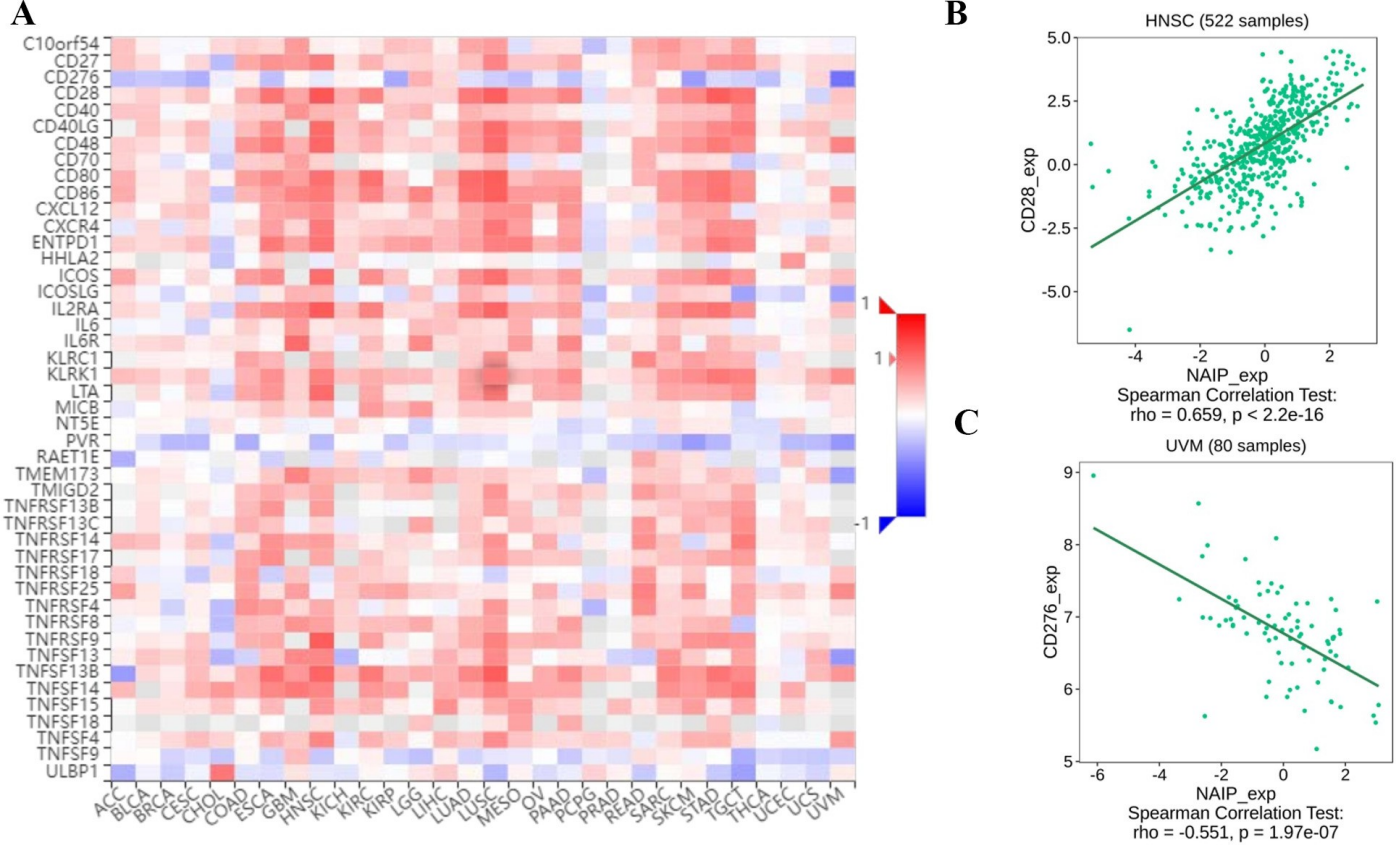
The expression correlation between NAIP and immune stimulators. Red indicates positive correlation whereas blue indicates negative correlation. (A) Dotplot of the strongest positive association. (B) Dotplot of the strongest negative association.

**Fig 11:**
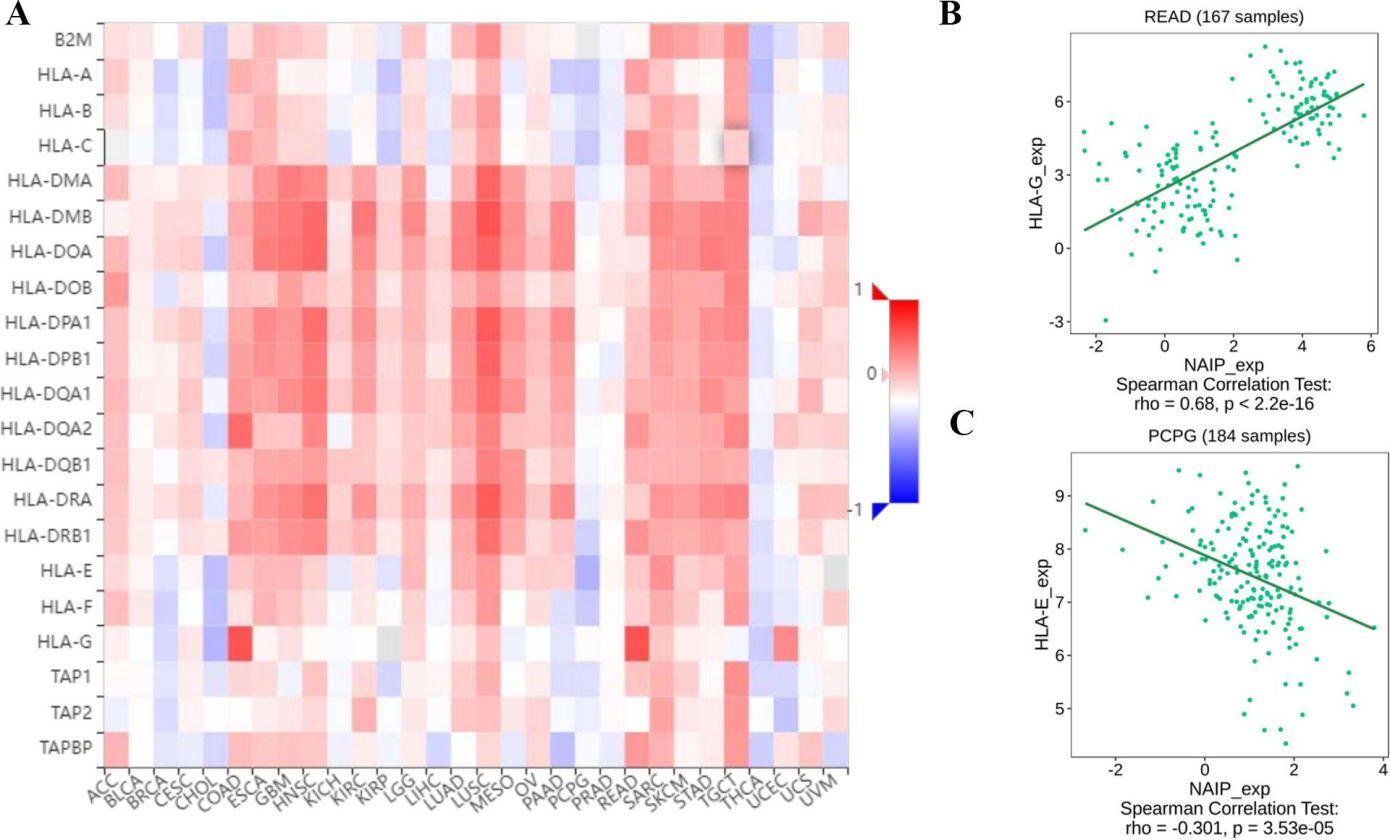
The expression correlation between NAIP and MHC molecules. Red indicates positive correlation whereas blue indicates negative correlation. (A) Dotplot of the strongest positive association. (B) Dotplot of the strongest negative association.

Fig 8 demonstrates the tumor immune microenvironment (including immune score and stromal score) and immune cell infiltration. NAIP expression was positively correlated with the ESCA, GBM, HNSC, LUAD, LUSC, SARC, and STAD immune score, and with the LAML, LUSC, and PAAD stromal score. For immune cell infiltration, NAIP expression was negatively correlated to B cells naïve (LAMA) and activated dendritic cells (ESCA). In LAML, NAIP expression was positively correlated to monocytes and macrophages M2. As shown in Fig 9, among 24 types of immune inhibitors, NAIP levels were positively correlated with CSF1R in HNSC, and negatively related to PVRL2 in UVM. Furthermore, 45 immune stimulators were analyzed (Fig 10), indicating that NAIP levels were positively correlated to CD28 in HNSC and negatively correlated to CD276 in UVM. The expression correlation between NAIP and MHC molecules showed that NAIP levels were positively correlated with HLA-G in READ and negatively associated with HLA-E in PCPG (Fig 11).

### TMB and MSI

**Fig 12:**
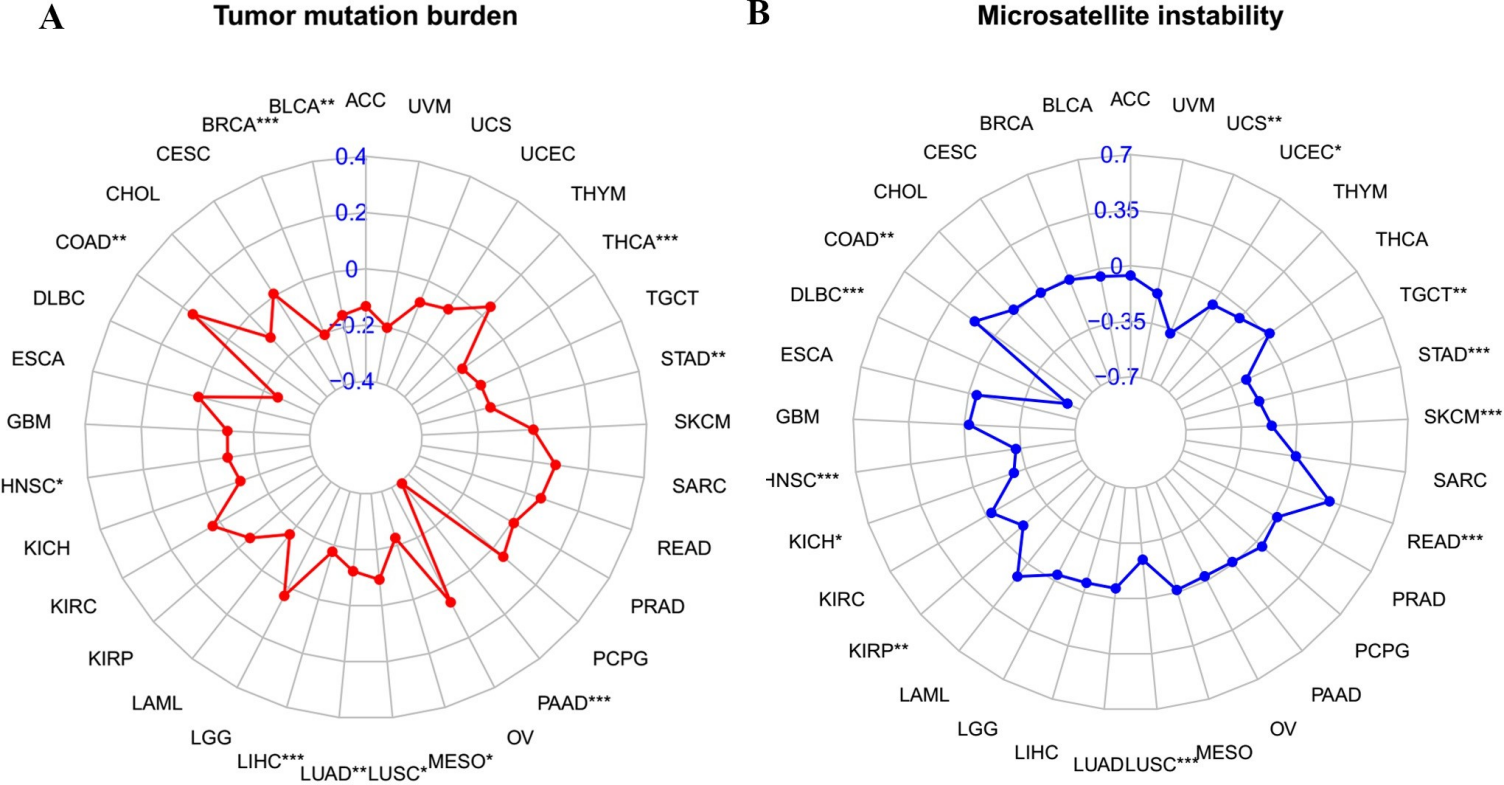
The correlation between NAIP and both TMB and MSI. (A) The Rader chart showing correlation between expression levels of NAIP and TMB. (B) The Rader chart showing correlation between expression levels of NAIP and MSI.

The relationship between NAIP and TMB/MSI revealed that NAIP levels were remarkably associated with TMB in BRCA, BLCA, THCA, STAD, PAAD, MESO, LUSC, LUAD, LIHC, HNSC, and COAD (Fig 12A). In COAD, high NAIP expression was positively correlated to TMB, suggesting better prognosis and potential for immunotherapy. In terms of MSI, the NAIP levels were positively correlated to COAD and READ and negatively associated with UCS, UCEC, TGCT, STAD, SKCM, LUSC, KIRP, KICH, HNSC, and DLBC (Fig 12B).

### PPI network and GSEA

**Fig 13:**
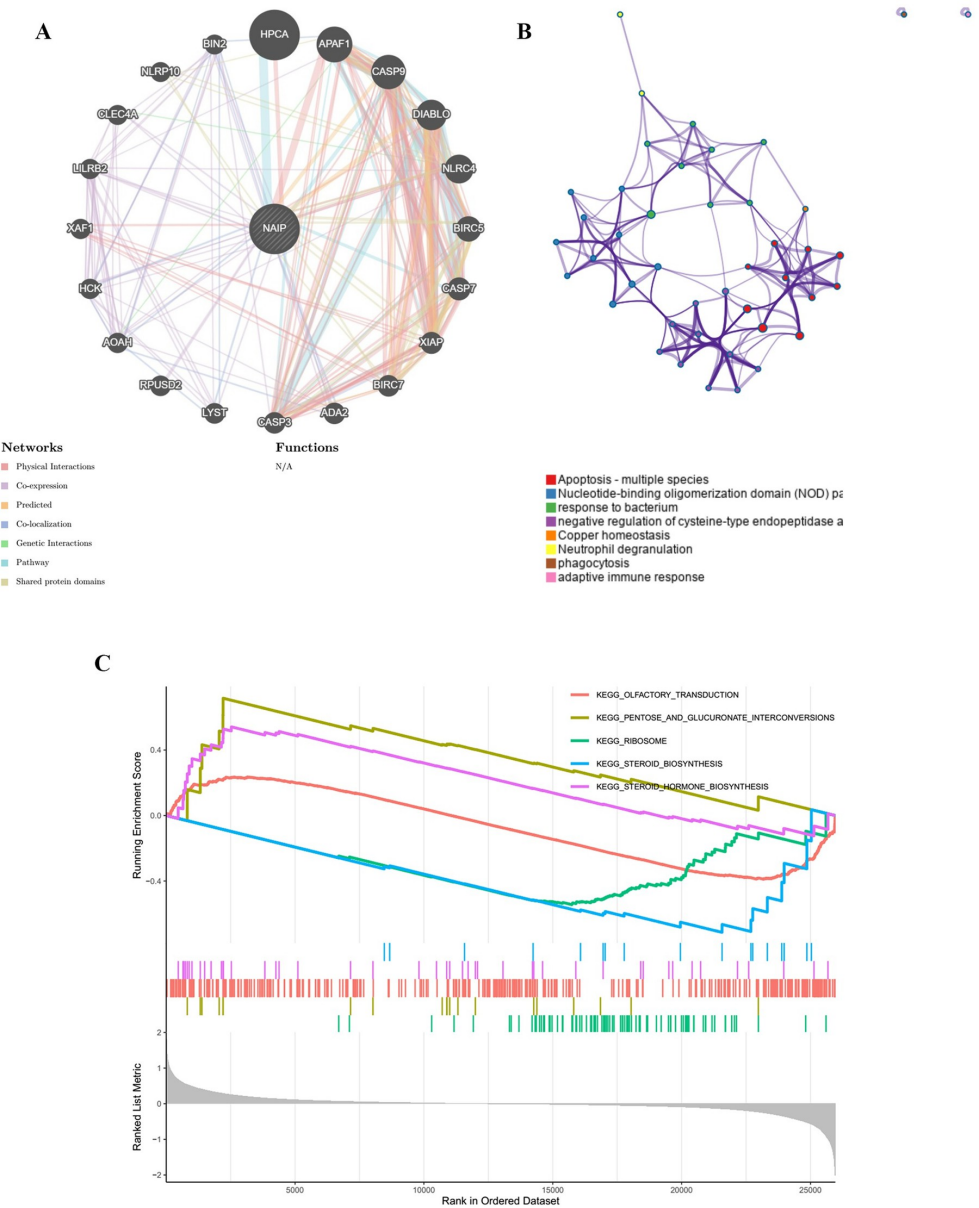
PPI network and enrichment analysis (GSEA). (A) Construction of a PPI network with 21 genes centered on NAIP. (B) Enrichment analysis using Matescape. (C) Gene set enrichment analysis (GSEA).

A PPI network of 21 genes centred on NAIP was performed with GeneMANIA (Fig 13A), which was then analyzed for enrichment using Metascape (Fig 13B). The outcomes indicated that these 21 genes were concentrated in apoptosis, the nucleotide-binding oligomerization domain (NOD) pathway, bacterial response, negative regulation of cysteine-type endopeptidase activity involved in apoptotic process, copper homeostasis, neutrophil degranulation, phagocytosis, and adaptive immune response. In addition, GSEA revealed that KEGG terms in the high NAIP expression were primarily enriched in pentose and glucuronate interconversions, olfactory transduction, and steroid hormone biosynthesis, while in the low NAIP expression were mainly enriched in ribosome and steroid biosynthesis (Fig 13C).

### Immunotherapeutic response of NAIP

**Fig 14:**
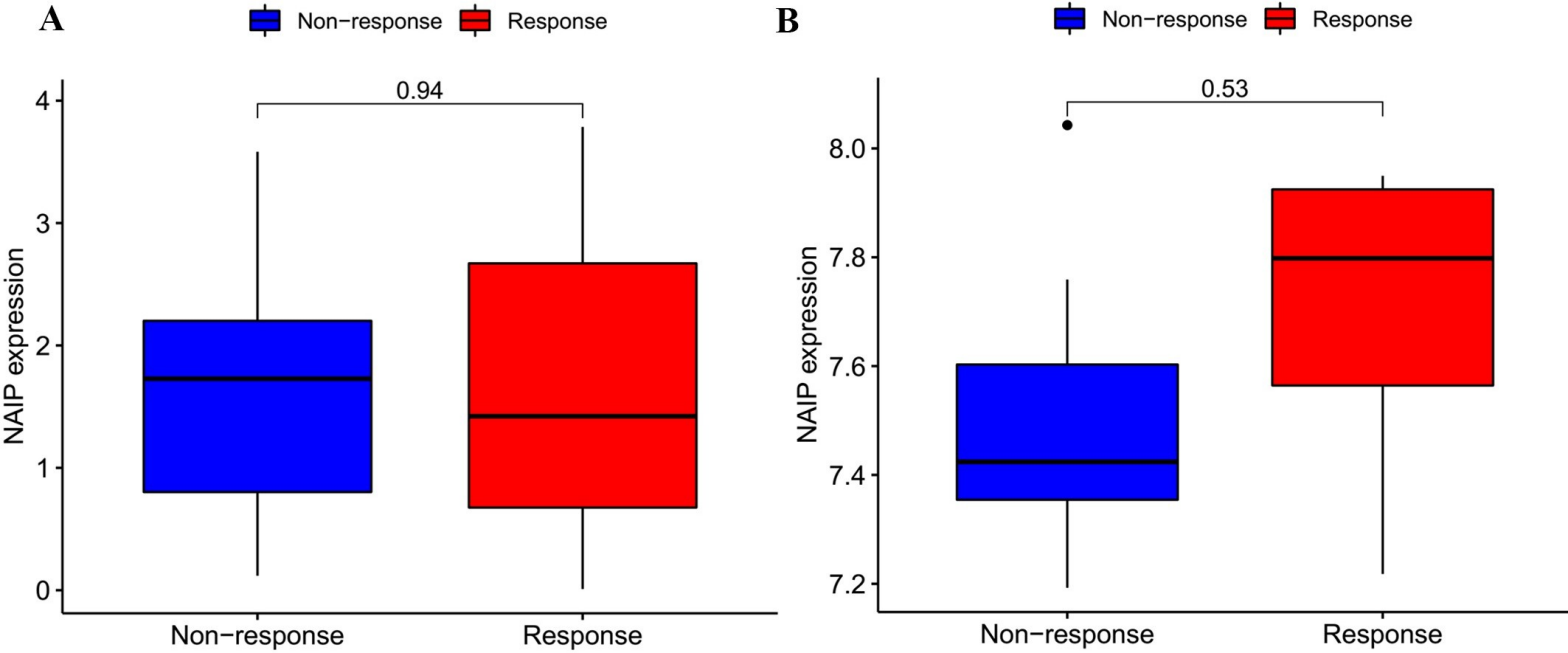
The correlation between NAIP and immunotherapeutic response. (A) GSE78220. (B) GSE67501.

As illustrated in Fig 14, there were no significant differences in NAIP expression between response and non-response groups in both independent cohorts.

## Discussion

In the current study, increased neutrophil infiltration was observed in IDD compared to controls. A remarkable increase in neutrophil accumulation has been reported with the development of IDD [34]. Besides, neutrophil-derived S100A8 is regarded as a therapeutic target for inflammation-related illnesses [35], while S100A8 have also been suspected of regulating neutrophil accumulation through CD11b upregulation [35]. These findings underline that targeting inflammation-dependent responses, such as neutrophil recruitment, could be an effective therapy in IDD patients.

We identified 273 significant DEGs by analyzing the GSE124272 dataset, and KEGG enrichment analysis primarily detected the PPAR signaling pathway and neutrophil extracellular trap formation. It has been revealed that PPAR -γ agonist pioglitazone protects against IL-17-induced intervertebral disc inflammation and degeneration via suppression of the NF-κB signaling pathway, which may provide a novel perspective on PPAR-γ agonist as a potential treatment for IDD [36]. GO enrichment analysis revealed that these genes were primarily associated with neutrophil degranulation and neutrophil activation involved in immune response, indicating that DEGs have mainly immune-related functions.

The most relevant blue module (containing 176 hub genes) w was chosen for additional investigation after WGCNA was used to identify IDD-related modules. Eight co-expressed genes (CLEC4E, NAIP, SLC11A1, FCGR1A, IL1R1, HSPA6, ARG1, and IL1R2) were subsequently discovered by intersecting 273 DEGs, 176 hub genes from the blue module, and 3178 immune-related genes. The present study identified NAIP as the key characteristic gene via overlapped genes acquired from the LASSO, SVM-RFE, and random forest algorithms. No previous research has reported the important role of NAIP in IDD now. Therefore, NAIP might influence the progression of IDD and could be utilized in the diagnosis of this disease. Currently, several articles have revealed that NAIP inflammasomes play a significant role in infectious diseases, auto inflammatory diseases, and cancer [37, 38]. Therefore, we hypothesized that NAIP may also play an essential part in IDD. In addition, the emerging role of NAIP in tumorigenesis can help identify novel pathway targets for the development of immunotherapies.

Furthermore, we found that the expression of NAIP increased in some carcinoma categories, indicating the possibility that NAIP could serve as an oncogene. Survival analysis also demonstrated the prognostic significance of NAIP. These findings suggest that targeted treatment of NAIP may improve the prognosis of patients in diverse carcinoma categories. A recent study indicated that NAIP could play a significant part in the inhibition of apoptosis through a reverse effect on caspases. Dysregulated NAIP may contribute to the occurrence of carcinoma and neurodegenerative disorders [39]. In breast carcinoma, p53 promotes the occurrence or development of breast cancer by negatively regulating NAIP expression [37]. NAIP has also been reported to be a potential molecular treatment target for hematological malignancies [40]. These results reveal that NAIP may provide a new target for anticancer treatment.

In addition, LAML showed that NAIP was strongly correlated to B Cells naïve, Macrophages M2, and Monocytes. In ESCA, dendritic cells were negatively associated with NAIP. Furthermore, the PPI indicated that these 21 genes were concentrated in apoptosis, NOD pathway and so on. Current literature also reports that deficiencies in apoptosis mechanisms and chronic activation of NOD receptors may lead to the development of various illnesses such as carcinomas. Also, the GSEA analysis revealed that high NAIP expression was primarily concentrated in pentose and glucuronate interconversions and steroid hormone biosynthesis, which were related to various carcinoma categories [41, 42].

Nevertheless, there were no significant differences between NAIP and immunotherapeutic reactions in the two cohorts. Since only two cohorts received immunotherapy, it is difficult to assess the actual immune response effect of NAIP. Therefore, more studies on immunotherapy ought to be carried out in the future.

To our knowledge, this is the first study that focuses on the roles of NAIP in 33 different cancer types and provides a novel perspective on the association between neoplastic and nonneoplastic diseases (IDD). However, the article also exists limitations. Firstly, our bioinformatic results are preliminary and need to be verified by further experiments. Secondly, further clinical experiments on cancer patients with IDD should be conducted to explore and validate the association between NAIP and cancer patients with IDD.

## Conclusion

The present study highlights that NAIP is a key immunity gene. Pan-cancer analysis revealed a noteworthy association between NAIP and prognosis and immune infiltration in diverse carcinoma categories, indicating that NAIP is a promising biomarker for cancer therapy. Given its effect on IDD and cancer, targeted treatment of NAIP may reduce the incidence of IDD. We are convinced that such discoveries will provide the basis for future research and clinical applications.

## Supporting information

S1 Table 33 types of human cancers involved in our study. (DOCX)
